# Isolation, characterization, and antibiotic resistance of *Vibrio* spp. in sea turtles from Northwestern Mexico

**DOI:** 10.3389/fmicb.2015.00635

**Published:** 2015-06-25

**Authors:** Alan A. Zavala-Norzagaray, A. Alonso Aguirre, Jorge Velazquez-Roman, Héctor Flores-Villaseñor, Nidia León-Sicairos, C. P. Ley-Quiñonez, Lucio De Jesús Hernández-Díaz, Adrian Canizalez-Roman

**Affiliations:** ^1^Programa Regional Para el Doctorado en Biotecnología, Facultad de Ciencias Químicas Biológicas, Universidad Autonoma de SinaloaCuliacán, México; ^2^Departamento de medio ambiente y desarrollo comunitario, Instituto Politécnico Nacional, Centro Interdisciplinario de Investigación para el Desarrollo Integral Regional - SinaloaGuasave, Mexico; ^3^Department of Environmental Science and Policy, George Mason UniversityFairfax, VA, USA; ^4^Research Unit, School of Medicine, Autonomous University of SinaloaCuliacán, Mexico; ^5^Pediatric Hospital of SinaloaCuliacán, Mexico; ^6^The Sinaloa State Public Health Laboratory, Secretariat of HealthCuliacán, Mexico

**Keywords:** antibiotic resistance, bacterial diversity, *Chelonia mydas agassizii*, *Lepidochelys olivacea*, Mexico, sea turtles, *Vibrio spp*.

## Abstract

The aerobic oral and cloacal bacterial microbiota and their antimicrobial resistance were characterized for 64 apparently healthy sea turtles captured at their foraging grounds in Ojo de Liebre Lagoon (OLL), Baja California Sur (BCS), Mexico (Pacific Ocean) and the lagoon system of Navachiste (LSN) and Marine Area of Influence (MAI), Guasave, Sinaloa (Gulf of California). A total of 34 black turtles (*Chelonia mydas agassizii*) were sampled in OLL and eight black turtles and 22 olive ridley turtles (*Lepidochelys olivacea*) were sampled in LSN and MAI, respectively from January to December 2012. We isolated 13 different species of Gram-negative bacteria. The most frequently isolated bacteria were *Vibrio alginolyticus* in 39/64 (60%), *V. parahaemolyticus* in 17/64 (26%), and *V. cholerae* in 6/64 (9%). However, *V. cholerae* was isolated only from turtles captured from the Gulf of California (MAI). Among *V. parahaemolyticu*s strains, six O serogroups and eight serovars were identified from which 5/17 (29.4%) belonged to the pathogenic strains (*tdh*^+^ gene) and 2/17 (11.7%) had the pandemic clone (*tdh*^+^ and *toxRS/new*^+^). Among *V. cholerae* strains, all were identified as non-O1/non-O139, and in 4/6 (66%) the accessory cholera enterotoxin gene (*ace*) was identified but without virulence gene *zot*, *ctxA*, *and ctxB*. Of the isolated *V. parahaemolyticus, V. cholerae*, and *V. alginolyticus* strains, 94.1, 33.4, and 100% demonstrated resistance to at least one commonly prescribed antibiotic (primarily to ampicillin), respectively. In conclusion, the presence of several potential (toxigenic) human pathogens in sea turtles may represent transmission of environmental microbes and a high-risk of food-borne disease. Therefore, based on the fact that it is illegal and unhealthy, we discourage the consumption of sea turtle meat or eggs in northwestern Mexico.

## Introduction

Sea turtles are air-breathing, marine reptiles of the order *Testudines*. The advances in their medical management, the studies on causes of morbidity and mortality during stranding events, and the efforts to conserve them, have increased in recent years. Despite these efforts, six of the seven species of sea turtles are classified as threatened or endangered by IUCN Red List of Threatened Species (IUCN, 2014). Sea turtles can be considered excellent sentinel species of marine ecosystem health due to their ecological and physiological characteristics, including long life spans, long period of time to reach sexual maturity, and high site fidelity to near-coastal feeding habitats (Aguirre and Lutz, 2004). Sea turtles appear to be highly susceptible to biological and chemical insults despite their robust appearance (Lutcavage et al., 1997). As with other marine vertebrate species, sea turtles are threatened by increasing anthropogenic activities including fisheries bycatch; illegal traffic of meat, eggs, and their parts; coastal development; various forms of plastic; global environmental change; and, environmental pollution (Aguirre and Lutz, 2004).

The presence of many contaminants in Northwestern Mexico is related to agricultural runoff. For example, methoxychlor, endrin, and heptachlor levels in the Navachiste-Macapule lagoon system suggested that these compounds were continuously applied although their use is forbidden (Montes et al., 2012). Several recent studies evaluating heavy metals and their potential impact on sea turtles in Northwestern Mexico have been reported (Frías-Espericueta et al., 2006; Ley-Quinonez et al., 2011; Ley-Quiñónez et al., 2013; Zavala-Norzagaray et al., 2014). The region encompasses the northern nesting and feeding distribution for black (*Chelonia mydas agassizii*) and olive ridley (*Lepidochelys olivacea*) turtles (Ley-Quiñónez et al., 2013; Aguilar-Gonzalez et al., 2014; Zavala-Norzagaray et al., 2014). The effects of heavy metals and other environmental contaminants in sea turtles have been previously documented as one of the potential synergic etiologies of marine turtle fibropapillomatosis (Aguirre et al., 1994, 2006; Lutcavage et al., 1997; Aguirre and Lutz, 2004). In addition, contaminant loads can increase the incidence of other diseases and could affect various functional processes (Camacho et al., 2013) representing a serious threat to dwindling sea turtle populations (Garcia-Fernandez et al., 2009).

Although there is no food safety microbiology for the consumption of sea turtle meat or eggs, this illegal practice is common in countries with coastal areas worldwide. The health effects of humans consuming sea turtles infected with zoonotic pathogens have been reported (Aguirre et al., 2006). For example, *Vibrio mimicus* in Costa Rica (Campos et al., 1996), *V. cholerae* in China (Lu et al., 2006), and *Salmonella chester* in Australia (O'grady and Krause, 1999) were associated to human disease by consumption of sea turtle meat and/or eggs. Therefore, it is important to effectively communicate accurate information regarding the potential human health hazards associated with the consumption of sea turtles and their eggs in areas where this practice is common (Aguirre et al., 2006).

Many bacteria have been identified as the cause of diseases in marine turtles kept in captivity (Chuen-Im et al., 2010; Arena et al., 2014). In addition, many of these bacteria may be pathogenic to humans (Warwick et al., 2013). Other bacteria including *Aeromonas hydrophila*, *V. alginolyticus*, *Pseudomonas fluorescens*, *Flavobacterium* spp., and *Bacillus* spp. are common bacterial microbiota in sea turtles from Hawaii and Australia and are associated with other diseases such as ulcerative stomatitis, obstructive rhinitis-pneumonia complex and fibropapillomatosis (Glazebrook and Campbell, 1990; Glazebrook et al., 1993; Aguirre et al., 1994). Among these bacteria, *Vibrio spp*. are commonly found, naturally, in aquatic environments and can cause infections to humans (Chowdhury et al., 1989; West, 1989; Chakraborty et al., 1997). Particularly, *V. parahaemolyticus* infections have increased globally; they are usually associated with eating raw or undercooked sea products (Nair et al., 2007; Velazquez-Roman et al., 2012, 2014; Hernández-Díaz et al., 2015). To the best of our knowledge, there are no reports of the presence of the bacterial diversity linked to disease in sea turtles in Mexico.

The aim of this study was to identify, characterize, and determine antibiotic resistance of potentially pathogenic bacteria isolated from oral and cloacal swabs from black turtles and olive ridley turtles in northwestern Mexico.

## Materials and methods

### Study site

During January to December 2012, sea turtle surveys were conducted in selected feeding grounds in the states of Baja California Sur (BCS) and Sinaloa (SIN). Ojo de Liebre Lagoon (OLL) (27.7500° N, 114.2500° W) is located on the Pacific coast near the border between BCS and Baja California and it is part of the El Vizcaino Biosphere Reserve. Eelgrass (*Zostera marina*) and several species of benthic macroalgae are abundant in the lagoon (Lopez-Castro et al., 2010). The highly productive waters of BCS have been revered for decades and recognized for centuries for the abundance and diversity of charismatic megafauna they attract (Lopez-Castro et al., 2010; Micheli et al., 2012). SIN has 16 coastal lagoons with mostly surrounded by irrigation districts where commercial fisheries and aquaculture farms represent important economic activities (Hernández-Cornejo et al., 2005; Gonzalez-Farias et al., 2006; Aguilar-Gonzalez et al., 2014). The Navachiste-Macapule lagoon (25.4–25.7° N and 108.85–108.55° W) is a complex coastal system with an approximate area of 24,000 ha. It is located in the municipality of Guasave, in the southeast coast of the Gulf of California (Montes et al., 2012). This lagoon has a great ecological and economic importance, as it supports a variety of oyster (*Crassostrea virginica*), clam (*Protothaca staminea*), mullet (*Mugil cephalus*), mojarra (*Gerres cinereus*), puffer (*Arthron hispidus*), snapper (*Pagrus auratus*), jewfish (*Argyrosomus hololepidotus*), and snook (*Centropomus undecimalis*) fisheries, as well as intensive shrimp (*Penaeus vannamei*) aquaculture activities (Orduna-Rojas and Longoria-Espinoza, 2006; Aguilar-Gonzalez et al., 2014).

### Sample collection

Sea turtles were captured unharmed using fishing nets and snorkel equipment (Ley-Quiñónez et al., 2013) and transported to a “floating dock” for examination, sampling and determination of morphometric parameters. All turtles were released alive and unharmed. Two nasopharyngeal and cloacal swabs were collected from each turtle for microbiology. The swabs were placed in alkaline peptone water at pH 8.5 (APW) for *Vibrio* spp. and in buffered peptone water pH 7.2 (BPW) for *Enterobacteriacea*; then transported to the School of Medicine laboratory at the Autonomous University of Sinaloa for bacteriological isolation and identification.

### Isolation and identification of bacterial strains

For *Vibrio* spp., all the nasopharyngeal and cloacal swabs were placed in APW and streaked onto thiosulfate citrate bile salts sucrose agar (TCBS; Becton-Dickinson, USA), and CHROMagar *Vibrio*, (CHROMagar Paris, France). The plates were incubated overnight at 37°C. From each plate, green and yellow colonies in TCBS or blue and violet colonies in CHROMagar *Vibrio* exhibiting diverse morphology were transferred to TSA-2% NaCl agar for purity. These plates were incubated overnight at 37°C and proceeded with identification using a single isolated colony. Each colony was examined by using the oxidase test and all biochemical tests described in the Bacteriological Analytical Manual of the Food and Drug Administration for *Vibrio* (Kaysner and De Paola, 2004; Canizalez-Roman et al., 2011). At least three typical colonies of *V. parahaemolyticus* and *V. cholerae* were isolated from each plate and subjected to identification by biochemical test and PCR. After identification of *V. parahaemolyticus*, *V. cholera*, and *V. alginolyticus* a single colony from each sample was used to continue the analysis (serotyping, virulence genes or antibiotic susceptibility testing). For *Enterobacteriacea*, specimens were placed in BPW and streaked onto *Salmonella-Shigella*, Hektöen and McConkey agar (Becton-Dickinson, USA). The plates were incubated overnight at 37°C. The presumptive colonies were transferred to TSA agar for purity. These plates were incubated overnight at 37°C and proceeded with identification using a single isolated colony. Each colony was examined by using the biochemical test (*Citrobacter freundii, E. coli, Edwarsiella spp., Aeromonas, Plesiomonas*, *Morganella* or *Proteus*, and *Providencia*) described in the Bacteriological Analytical Manual of the Food and Drug Administration (Andrews and Jacobson, 2013; Andrews et al., 2014; Feng et al., 2014).

### PCR assays

PCR assays were performed in a 25 μL volume consisting of 1X GoTaq green master mix (Promega), primers targeting the *tl* gene, the pR72H plasmid and the *tdh* and *trh* genes, the *toxRS* and *orf8* pandemic marker genes for *Vibrio parahaemolyticus* as previously described (Velazquez-Roman et al., 2012; Hernández-Díaz et al., 2015). *Vibrio cholerae* O1 and O139 were further confirmed for the presence of VC, *rfbO1, O139*, genes, and the *ctxA, ctxB, zot*, and *ace* toxigenic genes (Albert et al., 1997; Sarkar et al., 2002; Di Pinto et al., 2005; Goel et al., 2007) and 0.5 μg of purified genomic DNA template, with the remaining volume consisting of molecular biology grade water. PCR was routinely conducted in a Thermal Cycler C1000 (Bio-Rad Laboratories, Hercules, California). Ten microliter aliquots of each amplification product were separated by electrophoresis in a 2% agarose gel. Ethidium bromide staining (0.5 mg/ml) allowed for the visualization of DNA fragments with a digital imaging system (model E1 logia 100 imaging system; Kodak). The sizes of the PCR fragments were compared against a 50-bp DNA ladder (Promega DNA step ladder). To further identify diarrheagenic *E. coli* strains within our *E. coli* isolates, a protocol of sequential multiplex, duplex and single PCR reactions was used according to a previously published protocol work (Canizalez-Roman et al., 2013).

### Serotyping

*V. cholerae* serotyping was performed by using *V. cholerae* O1–specific polyvalent rabbit antiserum and O139-specific polyvalent rabbit antiserum obtained from the National Institute of Epidemiological Reference (InDRE), Mexico. The microagglutination test was used to determine the serogroup O1, Ogawa and Inaba, and serogroup O139 as described in the Bacteriological Analytical Manual of the Food and Drug Administration for *Vibrio* (Kaysner and De Paola, 2004). Furthermore, serotyping of *V. parahaemolyticus* isolates was performed by using a commercially available *V. parahaemolyticus* antiserum test kit (Denka Seiken, Tokyo, Japan) with O1–O11 antisera and 71 K antisera according to the manufacturer's instructions. Briefly, strains were grown overnight at 37°C on LB agar containing 3% NaCl. A pool of colonies was suspended in 1 mL of saline and then split in two 500 μl aliquots. For serotyping, an aliquot was heated up to 121°C for 1 h for O serotyping; if the serotype could not be obtained, the bacterial lysate was heated for an additional hour and then used for O serotyping. The second aliquot was used for serotyping based on the K antigen.

### Antibiotic susceptibility testing

All isolates of *V. parahaemolyticus*, *V. cholerae*, and *V. alginolyticus* were tested for antimicrobial susceptibility by a standard disc diffusion method on Mueller–Hinton agar. The protocol was performed as follows: fresh cultures were inoculated into LB broth and incubated until they reached an optical density equal to a MacFarland 0.5 standard. Bacterial cultures were then plated onto Mueller–Hinton agar and, then antibiotic disks (BD BBL, Franklin Lakes, NJ) were placed in a sterile environment. The plates were incubated at 37°C for 18–20 h. The diameters (in millimeters) of clear zones of growth inhibition around each antimicrobial agent disks were measured using a precision digital caliper (Absolute, Mitutoyo, Japan). Each bacterial species was classified as Resistant (R), Intermediately Resistant (I), or Susceptible (S) according to guidelines developed by the Clinical Laboratory Standard Institute (CLSI, 2011). The following antibiotics sensi-disc (BD BBL, Sensi-Disc, Becton, Dickinson and Company, USA), with their concentrations given in parentheses, were tested including ampicillin (10 μg), tetracycline (30 μg), trimethoprim–sulfamethoxazole (1.25 μg/23.75 μg), chloramphenicol (30 μg), nalidixic acid (30 μg), ciprofloxacin (5 μg), ceftazidime (30 μg), gentamicin (10 μg), and cefotaxime (30 μg). The following *V. parahaemolyticus* strains were used as a control organism: ATCC 17802, (*tdh*^−^) and multidrug resistant strain 727 (Leon-Sicairos et al., 2009).

## Results

### Identification of bacterial isolates

**Figure 1:**
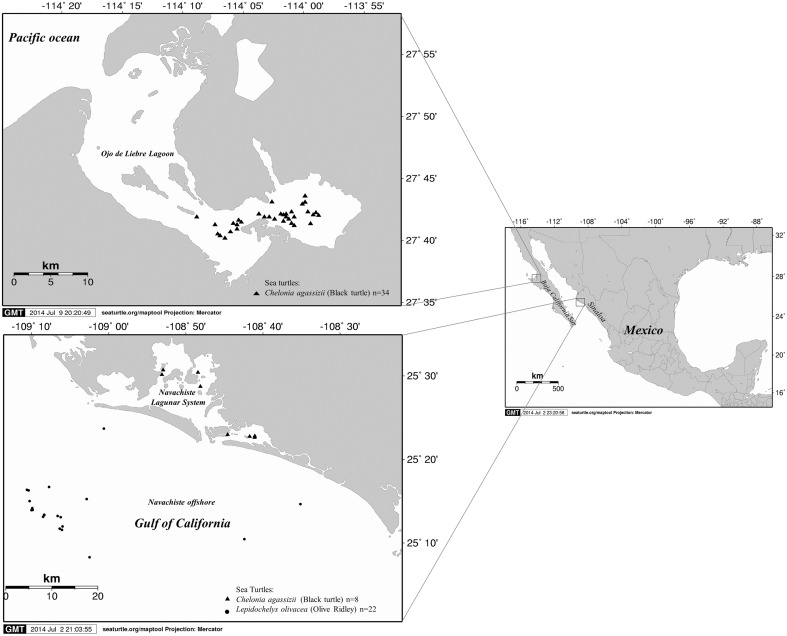
**Map of the study area and sites of capture of sea turtles, including in Ojo de Liebre Lagoon in Baja California Sur on the Pacific coast, the lagoon system of Navachiste and its influence zone in the Gulf of California in the state of Sinaloa**.

From January to December 2012, 34 black turtles (*C. mydas agassizii*) were sampled in OLL, and eight black and 22 olive ridley (*L. olivacea*) turtles were captured in LSN and MAI, respectively (Figure 1). A total of 82 bacterial isolates (42 from black turtles and 40 from olive ridley turtles) were identified (Table 1). According to the geographic site of sampling, 33 (40.3%) and 49 (59.7%) bacterial isolates from BCS and from SIN were detected, respectively (Table 1).

**Table 1:** **Bacterial species isolated from buccal cavity and cloacae of black turtles (*****Chelonia mydas agassizii*****) and olive ridley turtles (*****Lepidochelys olivacea*****)**.

**Bacteria**	**Anatomic isolation site**				**Sea turtles (*N* = 64)**		
	**Oral**		**Cloacal**		**BCS (*n* = 34)**	**Sinaloa (*n* = 30)**	
					***C. mydas agassizii n* = 34**	***C. mydas agassizii n* = 8**	***L. olivacea n* = 22**
*Aeromonas or Plesiomonas*				✔	2 (6%)	–	–
*Citrobacter*			✔		2 (6%)	–	–
*Citrobacter freundii*				✔	–	–	2 (9%)
*E. coli*				✔	1 (3%)	–	2 (9%)
*Edwarsiella spp*.	✔				–	–	1 (5%)
*Morganella or Proteus*			✔		2 (6%)	–	–
*Providencia*				✔	2 (6%)	–	–
*Vibrio alginolyticus*	✔			✔	21 (62%)	6 (75%)	12 (55%)
*Vibrio cholerae*		✔		✔	–		6 (27%)
*Vibrio fluvialis*				✔	–	1 (13%)	1 (5%)
*Vibrio furnisii*	✔				–	–	1 (5%)
*Vibrio parahaemolyticus*		✔	✔		1 (3%)	2 (25%)	14 (63%)
*Vibrio spp*.		✔			2 (6%)	–	1 (5%)

*BCS, Baja California Sur*.

Nine different microorganisms were isolated from olive ridley turtles including *Citrobacter freundii*, *E. coli*, *Edwarsiella* spp., *V. alginolyticus*, *V. cholerae*, *V. fluvialis*, *V. furnisii*, *V. parahaemolyticus*, and *Vibrio* spp. Eight different microorganisms were isolated from black turtles in BCS including *Aeromonas* or *Plesiomonas*, *Citrobacter*, *E. coli*, *Morganella*, and *Proteus*, *Providencia*, *V. alginolyticus*, *V. parahaemolyticus*, and *Vibrio* spp. Only three microorganisms were isolated from black turtles including *V. fluviales*, *V. alginolyticus*, and *V. parahaemolyticus* in Sinaloa (Table 1).

Overall, the predominant isolates in descending order of frequency were: *V. alginolyticus* (47.5%, 39/82), *V. parahaemolyticus* (20.7%, 17/82), *V. cholerae* (7.3%, 6/82) and other bacteria (25.6%, 21/82) (Table 1). *Vibrio alginolyticus* and *V. parahaemolyticus* were isolated in 62% (21/34) and 3% (1/34) from black turtles captured in BCS, respectively. However, in SIN, *V. alginolyticus* were isolated in 75% (6/8) and 55% (12/22), and *V. parahaemolyticus* were isolated in 25% (2/8) and 63% (14/22) from black turtles and olive ridleys, respectively. Interestingly, *V. cholerae* was only isolated in 27% (6/22) of olive ridley turtles (Table 1).

### Serovars and detection of virulence genes

*V. parahaemolyticus* was screened for the presence of virulence genes and serovars. There were 6 O serogroups, 4 different K types, and 8 serovars that could be identified in the 17 strains recognized or not by both O and K currently available antisera in *V. parahaemolyticus* isolates, (Table 2). A total of 47% (8/17) of the strains were not recognized by O antisera, while 82% (14/17) were not recognized by K antisera, and eight of these latter strains did not react to O:K antisera (OUT:KUT). Serogroups O1, O2, O4, O5, and O10 were identified. In BCS, one serotype OUT:KUT was isolated from black turtles. In Sinaloa, serotypes recognized were O1:KUT, O4:K10, O5:KUT, O10:KUT, OUT:KUT, O1:K32, O2:KUT, and O4:K13, isolated from olive ridleys and OUT:KUT, isolated from olive ridley and black turtles (Table 2).

**Table 2:** **Serological and virulence gene characteristics of** ***Vibrio parahaemolyticus*** **and** ***Vibrio cholerae***.

**Sea turtle species**	***Vibrio parahaemolyticus***						**Geographic zone**
	**Serovar**	**Total no. of isolates**	**Presence of each virulence gene**				
			***tdh***	***trh***	***Orf-8***	***toxRS/new***	
*C. mydas agassizii*	OUT:KUT	1	+	+	−	+	BCS
		2	−	−	−	−	SIN
*L. olivacea*	O1:KUT	1	−	−	−	−	SIN
	O1:K32	1	−	−	−	−	
	O2:KUT	1	+	−	−	−	
	O4:K10	1	+	−	−	+	
	O4:K13	1	−	−	−	−	
	O5:KUT	1	−	−	−	−	
		1	+	−	−	−	
	O10:KUT	2	+	−	−	−	
	OUT:KUT	4	−	−	−	−	
		1	+	−	−	−	
	***Vibrio cholerae***						
	**Serovar**	**Total no. of isolates**	**Presence of each virulence gene**				
			***ctxA***	***ctxB***	***zot***	***ace***	
*L. olivacea*	non-O1/non-O139	2	−	−	−	−	SIN
		4	−	−	−	+	

*+, Presence; −, absence*.

Based on the presence or absence of virulence genes, the *V. parahaemolyticus* isolates were classified into three groups: pandemic (*tdh*^+^, *toxRS/new*^+^, and/or *orf8*^+^), pathogenic (*tdh*^+^ and/or *trh*^+^), and non-pathogenic strains (*tdh*^−^ and *trh*^−^). Among *V. parahaemolyticus* strains, two strains (11.7%) were identified as pandemic isolates. One of these strains belonged to serotype OUT:KUT and carried the *tdh*, *trh*, and t*oxRS/new* genes (isolated from black turtles in BCS), whereas one pandemic O4:K10 strain carried the *tdh* and *toxRS/new* genes (isolated from olive ridley turtles in Sinaloa). A total of 29.4% (5/17) of isolates carried the virulence *tdh* gene and therefore were considered pathogenic strains (Table 2). Approximately 58.8% (10/17) of *V. parahaemolyticus* isolates were non-pathogenic.

Serological characterization of the six *V. cholerae* strains isolated from olive ridley turtles in Sinaloa, revealed that all strains belonged to the non-O1 and non-O139 serogroup. PCR studies revealed that four of the six isolates of *V. cholerae* non-O1/non-O139 harbored the *ace* gene; however, all strains tested were negative for the *ctxA*, *ctxB*, and *zot* genes (Table 2).

### Antibiotic resistance profiles

Antimicrobial susceptibility testing of *V. parahaemolyticus* isolated shown that all strains were resistant to ampicillin (94.1% resistant and 5.9% intermediate resistance), but >82% of isolates demonstrated susceptibility to tetracycline (88.2%), ceftazidime (82.3%) and chloramphenicol (88.2), and >64% to gentamicin and cefotaxime (Table 3). There were no strains resistant to ciprofloxacin, nalidixic acid, and sulfamethoxazole/trimethoprim (SXT) although inter-mediate zone sizes were observed in 23.5, 41.2, and 17.7% of the strains, respectively. Regarding overall antibiotic resistance, most *V. parahaemolyticus* strains (94.1%; 16/17) were non-susceptible to at least one antibiotic and 35.3% (6/17) of strains were resistant to two or more drugs (multidrug-resistant) (Table 3).

**Table 3:** **Antibiotic resistance among** ***Vibrio parahaemolyticus, Vibrio cholerae, and Vibrio alginolyticus*** **strains isolated from black turtles and olive ridley turtles**.

**Class and antimicrobial**	***Vibrio parahaemolyticus* No. (%)**				***Vibrio cholerae* non-O1/non-O139 No. (%)**				***Vibrio alginolyticus* No. (%)**			
	***n* = 17**	**^a^S**	**^b^I**	**^c^R**	***n* = 6**	**^a^S**	**^b^I**	**^c^R**	***n* = 39**	**^a^S**	**^b^I**	**^c^R**
**AMINOGLYCOSIDE**												
Gentamicin		11 (64.7)	4 (23.5)	2 (11.8)		4 (66.6)	1 (16.7)	1 (16.7)		7 (17.9)	17 (43.6)	15 (38.5)
**QUINOLONES AND FLUOROQUINOLONES**												
Ciprofloxacin		13 (76.5)	4 (23.5)	0		6 (100)	0	0		9 (23.1)	23 (59)	7 (17.9)
Nalidixic acid		10 (58.8)	7 (41.2)	0		5 (83.3)	1 (16.7)	0		26 (66.7)	11 (28.2)	2 (5.1)
*Sulfamethoxazole-trimethoprim*		14 (82.3)	3 (17.7)	0		6 (100)	0	0		23 (59)	13 (33.3)	3 (7.7)
**TETRACYCLINES**												
Tetracycline		15 (88.2)	0	2 (11.8)		6 (100)	0	0		36 (92.3)	0	3 (7.7)
**BETA LACTAMS**												
Ampicillin		0	1 (5.9)	16 (94.1)		3 (50)	2 (33.3)	1 (16.7)		5 (12.8)	0	34 (87.2)
**CEPHALOSPORINS**												
Ceftazidime		14 (82.3)	2 (11.8)	1 (5.9)		5 (83.3)	1 (16.7)	0		16 (41)	14 (35.9)	9 (23.1)
Cefotaxime		11 (64.7)	2 (11.8)	4 (23.5)		5 (83.3)	1 (16.7)	0		10 (25.6)	9 (23.1)	20 (51.3)
**PHENICOLS**												
Chloramphenicol		15 (88.2)	0	2 (11.8)		6 (100)	0	0		31 (79.4)	4 (10.3)	4 (10.3)
**NUMBER OF DRUGS RESISTANT TO:**												
0	1 (5.9%)				4 (66.6%)				0			
1	10 (58.8%)				2 (33.4%)				12 (30.8%)			
2	4 (23.5%)				0				8 (20.5%)			
3	0				0				12 (30.8%)			
4	1 (5.9%)				0				4 (10.3%)			
5	1 (5.9%)				0				1 (2.5%)			
6	0				0				2 (5.1%)			

a Susceptible,

b Intermediate,

c Resistant.

*Vibrio parahaemolyticus strains with resistance to 3, 6, 7, 8, and 9 drugs were not detected. Vibrio cholerae no-O1 strains with resistance to 2, 3, 4, 5, 6, 7, 8, and 9 drugs were not detected. Vibrio alginolyticus strains with resistance to 0, 7, 8, and 9 drugs were not detected*.

All strains of *V. cholerae* isolated were susceptible to ciprofloxacin, SXT, tetracycline, and chloramphenicol (Table 3). There were no strains resistant to nalidixic acid, ceftazidime, and cefotaxime although inter-mediate zone sizes were observed in 16.7% of the strains for each antibiotic. Low resistance (16.7%) was observed for gentamicin and ampicillin. In relation to antibiotic resistance, only the 33.4% (2/6) of strains were resistant to one antibiotic (Table 3).

On the other hand, almost all the *V. alginolyticus* strains (87.2%) exhibited resistance to ampicillin antibiotic, but low resistance was observed for tetracycline (7.7%), chloramphenicol (10.3%), nalidixic acid (5.1%), and to SXT (7.7%) (Table 3). There were no strains inter-mediate zone sizes observed to tetracycline and ampicillin. A high proportion of resistance and intermediate resistance were noticed among *V. alginolyticus* strains to gentamicin (82.1%), ciprofloxacin (76.9%), and cefotaxime (74.4%). Regarding overall antibiotic resistance, all *V. alginolyticus* strains were non-susceptible to at least one antibiotic and 69.2% (27/39) of strains were resistant to two or more drugs (multidrug-resistant). The resistance to 3–6 antibiotic was observed in 19 strains representing 48.7% (Table 3).

## Discussion

Sea turtles are long-distance, migratory animals and occupy niches in different marine environments and geographical regions throughout their different life cycle stages, usually ranging from pelagic environments, as hatchlings, to several coastal areas in their juvenile and adult stages. Due to migratory habits, sea turtles are susceptible to threats in both offshore and coastal environments (Bolten, 2003). Five of the world's seven sea turtle species occur along the Pacific coast of Mexico, making this region very important from a biological and socioeconomic point of view (Senko et al., 2009, 2011; Aguilar-Gonzalez et al., 2014). Black turtles that have grown large enough to reside in benthic environments have a nearly exclusive herbivorous diet consisting of selected macroalgae and sea grasses. They are found in the Mexican Pacific during all life history stages and the coastal waters of the eastern Pacific and Gulf of California provide important feeding and developmental habitats (Cliffton and Felger, 1982; Seminoff et al., 2002a,b; Senko et al., 2009, 2011; Aguilar-Gonzalez et al., 2014). Olive ridley turtles have a large range within the tropical and subtropical regions in the Pacific and Indian Oceans as well as the Southern Atlantic Ocean. This species spends most of its time within 15 km of shore, preferring shallow seas for feeding and sunbathing; however, this species is also observed in the open ocean (Eckert et al., 1999).

In Mexico, sea turtles have traditionally been an important resource for many coastal communities for centuries and have been used throughout the region for food, medicine, and decoration especially in BCS and SIN (Senko et al., 2009, 2011; Aguilar-Gonzalez et al., 2014). Coastal communities that consume sea turtles generally utilize the entire animal. While turtle meat is eaten directly (on the grill or stew), internal organs such as kidney and liver are used for soup. Oil is extracted from the fat as a cure for respiratory problems, especially in children, and eggs and blood are drunk raw as a remedy for anemia and asthma, and are valued as an aphrodisiac (Spotila, 2004; Delgado, 2005; Aguirre et al., 2006; Lohmann and Lohmann, 2006; Mancini and Koch, 2009).

The human impacts on the world's oceans have devastated populations, species, and ecosystems at a rapid scale (Aguirre et al., 2002; Aguirre and Lutz, 2004; Aguirre and Tabor, 2008). There are several zoonotic agents spilling over from terrestrial reservoirs to marine species and, on the other hand, zoonotic pathogens spill back to humans and domestic animals with severe consequences to wildlife health. Several bacterial species have been isolated from sea turtles, including *Salmonella*, *Mycobacterium*, *Vibrio*, and *E. coli*, which have been identified as potentially pathogenic to humans (Raidal et al., 1998; O'grady and Krause, 1999; Orós et al., 2005; Lu et al., 2006). In our study, a total of 82 bacterial isolates were identified including *C. freundii, E. coli, Edwarsiella spp., V. alginolyticus, V. cholerae, V. fluvialis, V. furnisii, V. parahaemolyticus*, and *Vibrio spp*. *Aeromonas* or *Plesiomonas*, *Morganella* or *Proteus*, *and Providencia*. All of these bacteria have been considered to be potentially pathogenic and opportunistic in sick sea turtles (Aguirre and Lutz, 2004; Orós et al., 2004, 2005). Besides, the predominant isolates from sea turtles were, *V. alginolyticus*, *V. parahaemolyticus*, *V. cholerae*, which are considered pathogenic to human health.

*V. parahaemolyticus*, are halophilic, Gram negative bacteria, that naturally inhabit marine and estuarine environments (Gutierrez West et al., 2013; Haley et al., 2014). The pathogen has emerged as a worldwide pandemic causing gastroenteritis related to consumption of sea products in recent years (Nair et al., 2007; Velazquez-Roman et al., 2014). Also, it has been demonstrated that the existence of the *tdh* and/or *trh* gene in a strain is associated with its ability to cause gastroenteritis (Nishibuchi and Kaper, 1995). Interestingly, in SIN, we have reported the presence of these bacteria in environmental samples and invertebrates (seawater, sediment, and shrimp) as well as fecal samples, identifying a high serodiversity and prevalence of pathogenic (*tdh*^+^) and pandemic (O3: K6, *tdh*^+^, and *toxRS/new*^+^) strains (Velazquez-Roman et al., 2012; Hernández-Díaz et al., 2015). However, in this study, we identified a novel serovar (04:K13) and the serotype O4:K10 with pandemic features in a different area from our previous investigations conducted during 2004–2013 (Velazquez-Roman et al., 2012; Hernández-Díaz et al., 2015). These novel serovars were isolated from olive ridley turtles, suggesting that this species could contribute to *V. parahaemolyticus* clones migrations in several ecosystems.

Importantly, olive ridley turtles nest from BCS (Lopez-Castro et al., 2004) to Peru (Kelez et al., 2009) with nesting reported from July to March. The scientific communities are coming to the conclusion that ballast discharge, global trade, and climate change represent the major underlying mechanisms for the global spread of pandemic *V. parahaemolyticus*, particularly clone O3:K6 (Velazquez-Roman et al., 2014). This spreading of pandemic *V. parahaemolyticus* is still a speculative question that requires further investigation since the pandemic and pathogenic strains can potentially migrate through sea turtles from Mexico to Peru or others countries including the United States. Due to the wide geographic distribution that sea turtles cover during their life cycle, they can serve as meaningful “sentinels” for overall ecosystem health (Aguirre and Lutz, 2004), and because of this, it is especially important to document and understand any factors that might affect the dissemination of pathogens.

To date, ca. 200 serogroups of *V. cholerae* have been recorded, and two (O1 and O139) have been associated with major cholera epidemics. The other serogroups, referred as non-O1/non-O139, have not been associated with epidemics but rather can cause sporadic diarrhea and occasional outbreaks (Chatterjee et al., 2009). In this study, *V. cholerae* was detected in six (9%) specimens. All isolates were non-O:1/non-O:139 serotypes and negative for *ctxA*, *ctxB*, and *zot* gene, but positive for *ace* gene by PCR. In China, turtles and their breeding environment have been reported major reservoirs of *V. cholerae* and responsible for many cholera outbreaks (Liu et al., 2006; Lu et al., 2006; Chang et al., 2007). In Zhejiang Province, the incidence of O1 serogroup of *V. cholerae* was found to be high (9%) in turtles and cholera epidemics in this region are associated with consumption of infected turtles (Lu et al., 2006). Identification of the non-O1/non-O139 serogroups of *V. cholerae* carrying virulence genes (*ac*e gene) in olive ridley turtles is very important since these new toxigenic strains with epidemic potential may emerge in the future if illegal consumption continues.

*V. alginolyticus* is pathogenic to a wide range of marine life including fish, mollusks, crustaceans, cnidarians, and sea turtles (Orós et al., 2004, 2005). In our study, *V. alginolyticus* were the predominant isolates from sea turtles and has been associated as an opportunistic pathogen for sea turtles in exudative bronchopneumonia and/or granulomatous pneumonia, traumatic skin lesions, granulomatous nephritis, renal abscesses, and necrotizing and/or granulomatous hepatitis and have been considered important causes of mortality among sea turtles (Orós et al., 2004, 2005). To the best of our knowledge, the present study is the first record of especially of *V. parahaemolyticus, V. cholerae*, and *V. alginolyticus* in black and olive ridley turtles in BCS and SIN, Mexico.

Another important contribution of this study was the investigation of susceptibility, or resistance of the isolated *V. parahaemolyticus*, *V. cholerae*, and *V. alginolyticus* strains to first-line antibiotics utilized in the region. All *V. parahaemolyticus* isolates were resistant to ampicillin, which was not a surprise as non-susceptibility to ampicillin is very common in *V. parahaemolyticus* strains isolated from environmental and clinical samples (Okuda et al., 1997; Wong et al., 2000; Roque et al., 2001; Sun et al., 2013; Letchumanan et al., 2015). We detected 23.5% of resistance to cefotaxime in *V. parahaemolyticus* from sea turtles, a similar prevalence of resistance to cefotaxime (20%) has also been reported in strains isolated in Italy from shellfish and clinical samples (Ottaviani et al., 2013). A number of studies have been reported low (Ceccarelli et al., 2015) and high (Jagadeeshan et al., 2009) prevalence of *V. cholerae* non-O1/non-O139 strains resistant to numerous antibiotics, isolated from environmental samples. In this study, only a few of the *non-O1/non-O139 V. cholerae* isolates from sea turtles were resistant to ampicillin or gentamicin. Most of the isolates were sensitive to all antibiotics tested.

All *V. alginolyticus* strains isolated in this study, were resistant to at least one antibiotic. Several studies reported a wide range of resistance for *V. alginolyticus* (Snoussi et al., 2008; Scarano et al., 2014). Although a high proportion of resistance and intermediate resistance were noticed to gentamicin (82.1%), ciprofloxacin (76.9%), and cefotaxime (74.4%). These results are in accordance with other studies which found high rates of resistance to gentamicin, ciprofloxacin, and cefotaxime (Snoussi et al., 2008; Lajnef et al., 2012).

Despite a federal ban on turtle hunting, consumption and trade in Mexico since 1990 (Gardner and Nichols, 2001; Senko et al., 2009; Aguilar-Gonzalez et al., 2014), sea turtles are captured by bycatch or incidentally mostly in the summer, precisely when consumption presents the greatest potential hazards to human health (Senko et al., 2009; Aguilar-Gonzalez et al., 2014). And when consumed, may have adverse human health effects, such as extreme dehydration, vomiting, diarrhea, and even death, due to the presence of bacteria, parasites, and environmental contaminants found in these animals (Aguirre et al., 2006; Senko et al., 2009; Aguilar-Gonzalez et al., 2014).

In conclusion, although fishermen from northwestern Mexico recognize that sea turtles might be contaminated and infected with potential pathogens, and that eating them could cause health problems (Aguirre et al., 2006; Senko et al., 2009; Aguilar-Gonzalez et al., 2014), they and their families continue consumption. In this study, we found the presence of several potential toxigenic and drug-resistant or multi-drug resistance human pathogens in sea turtles. Therefore, this information is important on possible health risks for humans in hope of behavioral changes that could benefit sea turtle conservation.

### Conflict of interest statement

The authors declare that the research was conducted in the absence of any commercial or financial relationships that could be construed as a potential conflict of interest.
